# Isolation, Purification and Structural Characterization of Two Novel Water-Soluble Polysaccharides from *Anredera cordifolia*

**DOI:** 10.3390/molecules22081276

**Published:** 2017-08-03

**Authors:** Zhi-Peng Zhang, Can-Can Shen, Fu-Li Gao, Hui Wei, Di-Feng Ren, Jun Lu

**Affiliations:** 1 Beijing Key Laboratory of Forest Food Processing and Safety, College of Biological Sciences and Biotechnology, Beijing Forestry University, 100083 Beijing, China; zhangzhipeng15@bjfu.edu.cn (Z.-P.Z.); C_canQ619@163.com (C.-C.S.); fuligao@bjfu.edu.cn (F.-L.G.); weihui_1990@126.com (H.W.); 2 Beijing Engineering Research Center of Protein & Functional Peptides, China National Research Institute of Food & Fermentation Industries, 100015 Beijing, China

**Keywords:** isolation, purification, polysaccharide, *Anredera cordifolia*, structural characterization

## Abstract

*Anredera cordifolia*, a climber and member of the Basellaceae family, has long been a traditional medicine used for the treatment of hyperglycemia in China. Two water-soluble polysaccharides, ACP1-1 and ACP2-1, were isolated from *A. cordifolia* seeds by hot water extraction. The two fractions, ACP1-1 and ACP2-1 with molecular weights of 46.78 kDa ± 0.03 and 586.8 kDa ± 0.05, respectively, were purified by chromatography. ACP1-1 contained mannose, glucose, galactose in a molar ratio of 1.08:4.65:1.75, whereas ACP2-1 contained arabinose, ribose, galactose, glucose, mannose in a molar ratio of 0.9:0.4:0.5:1.2:0.9. Based on methylation analysis, ultraviolet and Fourier transform-infrared spectroscopy, and periodate oxidation the main backbone chain of ACP1-1 contained (1→3,6)-galacturonopyranosyl residues interspersed with (1→4)-residues and (1→3)-mannopyranosyl residues. The main backbone chain of ACP2-1 contained (1→3)-galacturonopyranosyl residues interspersed with (1→4)-glucopyranosyl residues.

## 1. Introduction

Polysaccharides, which are made up of monosaccharides linked through glycosidic bonds, exist in plants, animals, and microorganisms. In recent years, some bioactive polysaccharides extracted and separated from plants, especially from medicinal plants, have attracted significant attention in the fields of pharmacology and biochemistry because of their potential biological activities, such as antioxidant [1,2], α-glucosidase inhibitory [3], anticoagulant [4,5], prebiotic [6], antitumor [7] and immunobiological effects [8]. Most polysaccharides derived from plants are relatively nontoxic, and any side effects are minimal compared with those of synthetic compounds [9,10]. Thus, increasing attention has been devoted to the study of bioactive polysaccharides and most of these studies performed on bioactive polysaccharides took traditional information on the use of medicinal plants and their related bioactivities as a guide to choose the plants that should be studied [11,12,13]. Meanwhile, many novel techniques such as ultrasound assisted extraction, ultrafiltration, centrifugation and pulsed electric energy have been applied to the extraction and purification of bioactive polysaccharides [14,15,16]. The search for efficient methods of extraction and purification of bioactive polysaccharides from plant has thus become a research hotspot.

*Anredera cordifolia* is a climbing succulent that is widely distributed in the southern parts of South America and China [17]. It belongs to the family Basellaceae, which includes 19 accepted species in four genera. In daily life, the tubers and leaves are used in traditional medicine applications as a sedative, supplement, and for the treatment of hyperglycemia [18]. In previous studies, *A*. *cordifolia* was reported to contain bioactive compounds that possess antibacterial, anti-obesity and anti-hypoglycemic, cytotoxic and anti-mutagenic, anti-diabetic, and anti-inflammatory activities [19]. Polysaccharides are found to be primary active components from many medicinal plants such as *Dendrobium* plants, *Astragalus membranaceus* and *Dimocarpus longan* Lour, which exert various pharmacological activities [20]. However, current studies have focused mainly on educts and their biological activities, and few reports describe the structural characteristics of polysaccharides extracted from *A. cordifolia*.

The biological activities of polysaccharides are correlated with their structural characterization. The type of monomer, linkage type and position, and the number and position of branches occurring within the polymer chain strongly influence the three-dimensional arrangement, and in addition to the molecular size, these factors determine polysaccharide behavior [21]. Physical properties, such as solubility, viscosity, and gelation, may also influence the biological activity because they can affect bioavailability [22,23]. Some studies suggested that polysaccharides have significantly different average molecular weights, however, fractions with similar monosaccharide compositions can display the same biological activity [24]. Therefore, the elucidation of molecular structures of polysaccharides occurring in medicinal plants is very important for predicting their biological behavior.

To our knowledge, there are no reports available on the structural elucidation of polysaccharides from *A. cordifolia*. Hence, the present study aimed to separate, purify, and structurally characterize water-soluble polysaccharides from *A. cordifolia*. The results could be used for further investigation of the structure-activity relationship and development of applications of *A. cordifolia* polysaccharides.

## 2. Results and Discussion

### 2.1. Isolation and Purification of Polysaccharides

**Figure 1:**
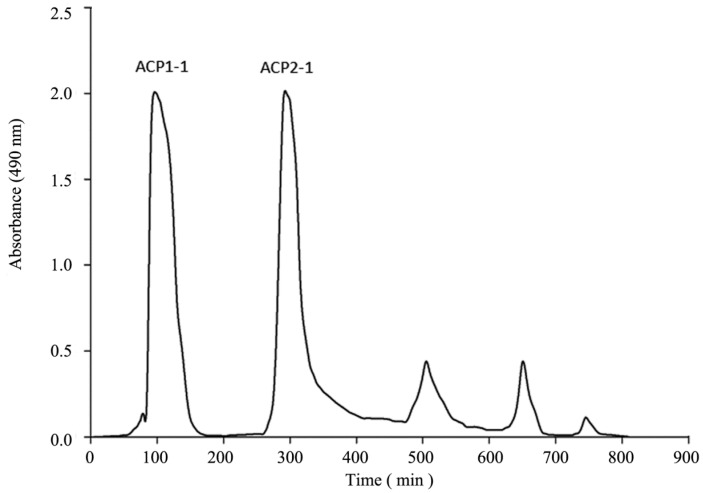
Elution profiles of crude polysaccharide ACP on a DEAE-52 column.

The crude polysaccharide ACP was obtained as a water-soluble white powder from *A. cordifolia* by hot-water extraction, ethanol precipitation, and deproteinization by the Sevag method, trichloroacetic acid method, and hydrochloric acid method. Decolorization was performed using five different macroporous resins. Among the three methods tested, the trichloroacetic acid method was the most effective approach to eliminate the protein, giving the highest protein clearance rate and polysaccharide retention rate among the tested methods. The hydrochloric acid method may cause partial hydrolysis of polysaccharides, resulting in reduced polysaccharide retention rate [25]. Other studies have reported that the efficiency of the Sevag method for the removal of proteins from *Ganoderma sinensis* was significantly lower compared with that of the trichloroacetic acid method, which is consistent with our results [26]. The protein clearance rate with the Sevag method was similar to that with the trichloroacetic acid method, but had only half of the polysaccharide retention rate of the trichloroacetic acid method (Table 1). There was a significant loss in the recovery of polysaccharides, which may be due to damage to the polysaccharides by the Sevag reagent as well as the co-removing effects of the glucoprotein during the harsh chemical treatment procedure [27]. As shown in Table 2, X-5 resins exhibited higher polysaccharide retention rates and decolorization rates than those of the other four resins. The low retention rates of polysaccharide on AB-8, D-101, HPD-400 and NKA-9 resins were probably a result of the high molecular weight and the high space structure of polysaccharides [28]. After deproteinizing and decolorizing, the polysaccharide content was 25.36%. The collected residues contained trace amounts of proteins and pigments. After submitting to condensation, precipitation, dialysis with water, and freeze-drying, ACP was further purified on the DEAE-cellulose A52 and Sephadex G-100 gel-filtration columns (Figure 1). The main fractions were collected, lyophilized, and named as ACP1-1 and ACP2-1 for further structural characterization.

### 2.2. Homogeneity and Molecular Weights of ACP1-1 and ACP2-1

The polysaccharides ACP1-1 and ACP2-1 each showed only one symmetrical and narrow peak with an elution time of about 21 min in the GPC/MALLS chromatograms, indicating that both of these polysaccharides are homogeneous. Based on calibration with standard dextrans, the average molecular weights of ACP1-1 and ACP2-1 were estimated to be about 46.78 kDa ± 0.03 and 586.8 kDa ± 0.05, respectively. The average molecular weight of ACP2-1 was 12 times that of ACP1-1, and was higher than the molecular weights reported for most other botanical polysaccharides, such as those isolated from *Allium macrostemon*, *Abies sibirica*, and *Lycium barbarum* [29,30,31]. The homogeneity was estimated by polydispersity index (Mw/Mn) and the Mw/Mn of ACP1-1 and ACP2-1 were 2.866 and 2.067, respectively. This indicated that the structure of ACP2-1 was more homogeneous than that of ACP1-1. Research has demonstrated that the higher molecular weight *Ziziphus jujuba* polysaccharide with a similar composition to that of the Jinsixiaozao polysaccharide, which displays higher antioxidant activities than the Jinsixiaozao polysaccharide because of its higher molecular weight, allowed the spatial conformation of the polysaccharides to be maintained [32].

### 2.3. Determination of the Monosaccharide Composition

**Figure 2:**
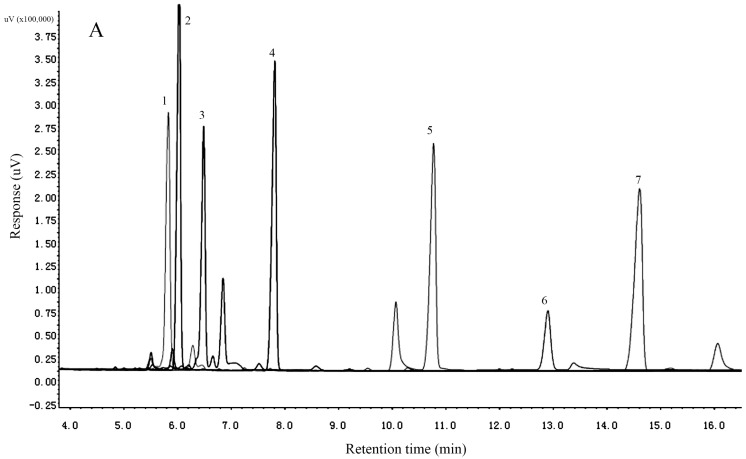

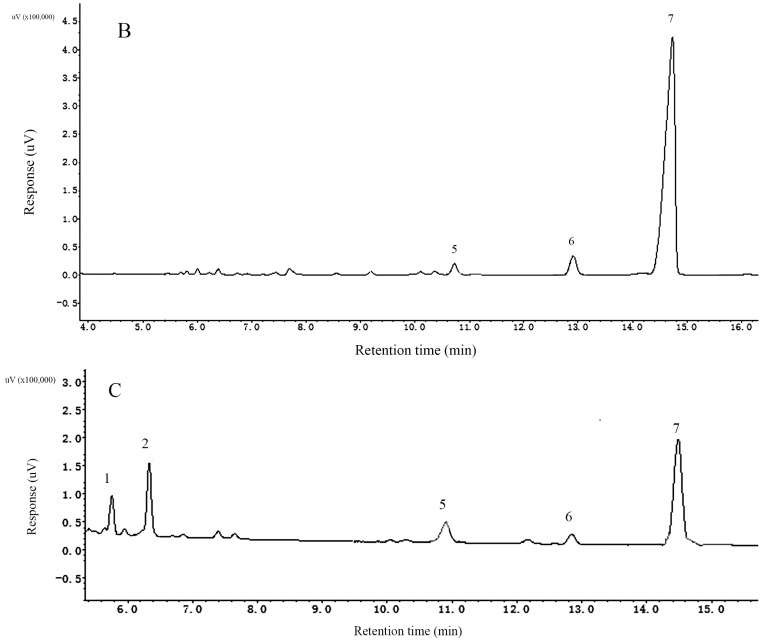
Gas chromatogram profiles of monosaccharide standards, ACP1-1 and ACP2-1. (**A**) Monosaccharide standards; (**B**) Monosaccharide composition of ACP1-1; (**C**) Gas chromatogram of monose compositions of ACP2-1. 1: arabinose; 2: rhamnose; 3: ribose; 4: xylose; 5: mannose; 6: galactose; 7: glucose.

Growing evidence suggests that the bioactivity of a polysaccharide is associated with its structure, including its monosaccharide composition, types of glycosidic linkages, and conformation [33]. The analysis of monosaccharide compositions commonly involves the cleavage of glycosidic linkages by complete acid hydrolysis, derivatization, and qualification and quantification with GC and HPLC [34]. The results indicated that ACP1-1 contained mannose, glucose and galactose in a molar ratio of 1.08:4.65:1.75, whereas ACP2-1 contained arabinose, ribose, galactose, glucose, and mannose in a molar ratio of 0.9:0.4:0.5:1.2:0.9 (Figure 2). In terms of peak area, glucose was the major monosaccharide in both ACP1-1 and ACP2-1. The results indicated that the monosaccharide composition of ACP2-1 was more complex than that of ACP1-1, and both of the polysaccharides were neutral. Comparison of our results with the literature showed good agreement with qualitative neutral polysaccharide composition [35]. Our results were in agreement with those for other medicinal and edible plants, such as *Acanthopanax sciadophylloides* and *Lycium barbarum*, which also contain glucose and galactose as abundant neutral sugars although in different molar ratios of 2:3 (*A. sciadophylloides*) and 1:5 (*L. barbarum*) [36,37]. Another study reported that a polysaccharide isolated from *G. capense* was a homopolysaccharide of glucose [38]. According to a recent report, glucose may be a fundamental monosaccharide in neutral polysaccharides from most medicinal plants and monosaccharide compositions that contained mannose, glucose and galactose generally possess antioxidant and immunomodulatory activity [20]. In this study, monosaccharide compositions of both ACP1-1 and ACP2-1 have mannose, glucose and galactose. The data indicated that ACP1-1 and ACP2-1 derived from *A. cordifolia* were a potential source of antioxidant and immunomodulatory activity suitable for use in functional food and therapeutic agents.

### 2.4. UV/Vis and FT-IR Analysis of the Polysaccharides

**Figure 3:**
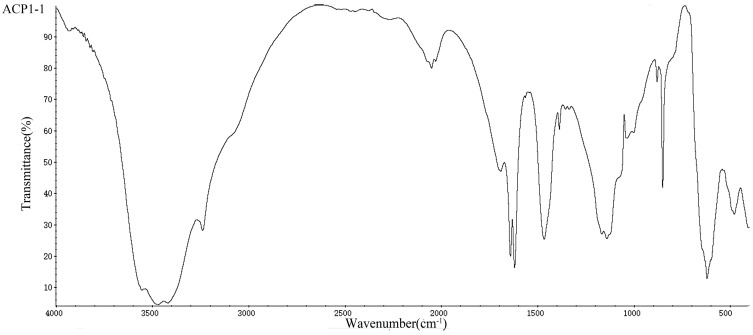

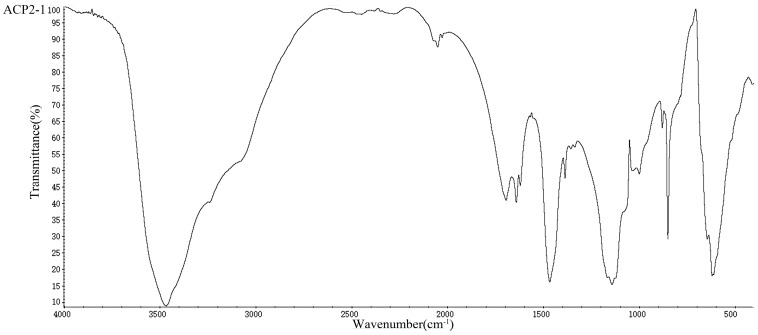
FT-IR spectrum of the ACP1-1 and ACP2-1 polysaccharide fraction.

The peak intensities at 280 nm and 260 nm in the UV/Vis spectra of ACP1-1 and APC2-1 were low, which indicated that they contained few protein and nucleic acid impurities. The various biological activities of polysaccharides are strongly related to their chemical compositions and configurations [39]. FT-IR spectroscopy is a powerful technique for the identification of characteristic organic groups in polysaccharides [40] and was used to analyze the structure of ACP1-1 and ACP2-1 in this study. The spectra (Figure 3) of the polysaccharides all displayed a broad peak between 3200 and 3600 cm^−1^, which was attributed to the stretching vibration of C-OH [41]. An intense peak between 1600 and 1700 cm^−1^ was attributed to the stretching vibration of C=O and the asymmetric stretching vibration of C=O [42]. An intense and broad band at around 1250–1550 cm^−1^ was attributed to C-H stretching vibrations, whereas a weak absorption band at about 550–700 cm^−1^ was attributed to O-H stretching vibrations [43,44]. Peaks between 1000 and 1200 cm^−1^ indicated the presence of C-O-C glycosidic bonds [45], and a weak absorption at around 849 cm^−1^ was attributed to a α-glycoside bond [46]. The characterization of ACP1-1 and ACP2-1 by FT-IR showed absorption bands typical of polysaccharides.

### 2.5. Periodate Oxidation

1 M glycosyl consumed 0.9975 mol of NaIO_4_ and produced 0.105 mol of HCOOH, which indicated non-reducing terminal residues or 1→6 linkages were present. The results showed that 0.35 mol of NaIO_4_ was consumed by ACP1-1, which indicated 1→2; 1→2,6; 1→4; 1→4, 6; or 1→3 linkages were present. Similarly, 0.21 mol of NaIO_4_ was consumed by ACP2-1, indicating the existence of 1→2; 1→4; or 1→3 linkages. A previous research indicated that the structural characteristics of polysaccharides, such as β-(1→3) linkages in the main chain, are important for their antitumor activities because they increase the activities of immunocompetent cells [47]. Moreover, polysaccharides containing 1→3 linkages bonds generally strengthen the immune system [48]. For example, Lentinan, α β-(1→3)-d-glucan isolated from *Lentinus edodes* is known for its potent anti-tumor and anti-viral activities since the 1970s, and it has been widely used as an alternative medicine [49].

### 2.6. Methylation Analysis

Methylation analysis has been used to determine the structure of carbohydrates for over a century and is still the most powerful method in determining the sugar linkages of polysaccharides [50]. The methylated products of ACP1-1 and ACP2-1 were analyzed by GC-MS. The fragmentation patterns of these peaks were identified and the molar ratios were estimated (Table 3 and Table 4). Seven fragments were detected for reduced ACP1-1, namely 2,4,6-Me3-Gal; 2,3,6-Me3-Glc; 3,6-Me2-Man; 2,3,4,6-Me4-Glc; 2,4,6-Me3-Man; 2,3,4-Me3-Gal; and 2,4-Me2-Man in a 5.02:38.12:2.49:1.30:1.12:1.52: 13.31 molar ratio. Two fragments were detected for reduced ACP2-1, namely 2,4,6-Me3-Gal and 2,3,6-Me3-Glc in a 1.10:9.87 molar ratio. The methylation results indicated that ACP1-1 mainly consisted of residues of (1→3)-galacturonopyranosyl, (1→4)-glucopyranosyl, (1→2, 4)-mannopyranosyl, non-reducing terminal, (1→3)-mannopyranosyl, (1→6)-galacturonopyranosyl, and (1→3,6)-manno-pyranosyl. The ratio of (1→4)-glucopyranosyl residues and (1→3, 6)-mannopyranosyl residues and (1→6)-galacturonopyranosyl residues were 1.30:1.12:1.52, suggesting that they could be joined together [51]. In addition, the presence of (1→3)- and (1→6)-linked Gal residues suggested that arabinogalactan type II (AG-II) might also be present in these neutral polysaccharides [52]. For ACP2-1, the methylation results indicated that it mainly consisted of residues of (1→3)-galacturono-pyranosyl, and (1→4)-glucopyranosyl. Moreover, (1→3)-galacturonopyranosyl residues and (1→4)-glucopyranosyl residues in a molar ratio of 1.10:9.87 suggested that every ten (1→3)-galacturonopyranosyl residues interspersed with one (1→4)-glucopyranosyl residues [51]. The molar ratios were in accordance with the overall monosaccharide composition described above. The monomers found for ACP1-1 and ACP2-1 were consistent with the results obtained from periodate oxidation. The methylation data suggested the main backbone chain of ACP1-1 was composed of (1→3, 6)-galacturonopyranosyl residues interspersed with (1→4)-glucopyranosyl residues and (1→3)-mannopyranosyl residues. Whereas the main backbone chain of ACP2-1 was composed of (1 →3)-galacturonopyranosyl residues interspersed with (1→4)-glucopyranosyl residues. To our knowledge, this is the first report on structural details of two novel water-soluble polysaccharides from *A. cordifolia*.

## 3. Materials and Methods

### 3.1. Plant Materials, Chemicals, and Instrumentation

Roots of *A. cordifolia* were purchased from Chinese herbal medicine planting cooperatives. The original plants were collected in October of 2015 at Hebei Province, China. The samples were freeze-dried and stored at −80 °C until required for further study. Diethylaminoethyl (DEAE)-cellulose A52 and Sephadex G-100 columns were purchased from Huamaike Biological Technology Co. (Beijing, China) Ultraviolet-visible (UV/Vis) spectroscopy was performed using an UltraViolet-4802 spectrometer (Shimadzu Enterprise Management Co., Guangzhou, China). Fourier transform-infrared spectroscopy (FT-IR) was performed on a Nicolet 6700 instrument (Thermo Fisher Scientific, Waltham, MA, USA). Gas chromatography-mass spectrometry (GC-MS) was carried out on a GC-201O gas chromatograph with a methyl polysiloxane capillary column (30 m × 0.25 mm, film thickness 0.25 mm), which was obtained from Shimadzu Enterprise Management Co. High-performance gel permeation chromatography (HPGPC) was performed using a Wyatt DAWN chromatograph (Wyatt Technology Corporation, California, USA). Pure monosaccharide standards of d-mannose (Man), d-ribose (Rib), d-arabinose (Ara), d-glucose (Glc) and d-galacturonic acid (GalA) with varying molecular weights (5000, 12,000, 25,000, 50,000, 80,000, 150,000, 270,000, 410,000, and 600,000 Da) were obtained from Humaike Biological Technology Co. Acetic acid, phenol, and trifluoroacetic acid (TFA) were purchased from Beijing Chemical Factory (Beijing, China). All other chemicals and reagents in this study were of analytical grade. Distilled water was used in all of the experiments.

### 3.2. Isolation and Purification of Polysaccharides

Air-dried powder of *A. cordifolia* (40 g) was extracted with 95% ethanol (1 L) at 90 °C in a water bath for 5 h under stirring to remove pigments, polyphenols, and monosaccharides. As the liquid cooled, it was centrifuged at 5000 rpm for 15 min at 4 °C. The above was performed three times, and the supernatants were combined, concentrated in a rotary evaporator under a reduced pressure, and then filtered. The concentrated solution was deproteinated by the Sevag method [53], trichloroacetic acid method [54], and hydrochloric acid method [55], with each method repeated six times. The solution was decolorized by the macroporous resin separation method. Hyperfiltration was applied to desalinate with a molecular weight membrane of 3 KDa and at a flow rate of 5.0 mL/min for 8 h. After that, the filtrate was precipitated by adding 99.5% ethanol (four times the volume of the aqueous extract) at room temperature for 8 h, and then centrifugation at 4000 rpm for 10 min at 4 °C. Finally, the precipitate was dissolved in distilled water and then lyophilized in a vacuum freeze dryer to obtain the crude polysaccharide. A sample of the polysaccharide (200 mg) was redissolved in distilled water, and then purified with the DEAE-cellulose A52 column (2.6 cm × 60 cm), which was equilibrated with distilled water. The polysaccharide was fractionated by stepwise elution with distilled water, followed by a gradient elution with aqueous NaCl (0–0.5 M) at a flow rate of 1.0 mL/min. Fractions (5 mL) were collected and the absorbance at 490 nm was measured using the phenol-sulfuric acid method [56]. The eluted solution was separated into two fractions (ACP1-1 and ACP2-1), which were further purified on the Sephadex G-100 gel filtration column (2.6 cm × 60 cm), and eluted with deionized water at a flow rate of 9 mL/h. The eluate was concentrated, dialyzed against water, and finally lyophilized to obtain white powders of the pure polysaccharides ACP1-1 and ACP2-1 for further study.

### 3.3. Determination of Homogeneity and Relative Molecular Weights

The homogeneity and molecular weights of ACP1-1 and ACP2-1 were determined by gel permeation chromatography and multi-angle laser light scattering (GPC/MALLS). The sample was diluted with ultrapure water and filtered through a 0.45 μm membrane on the GPC/MALLS instrument, and eluted with 0.1 M NaNO_3_ and 0.02% sodium azide at a flow rate of 0.5 mL/min. A refractive index detector was used for detection at 40 °C. Dn/Dc was 0.147 mL/g. The columns were calibrated with dextran T-series standards of known molecular weights (5000, 12,000, 25,000, 50,000, 80,000, 150,000, 270,000, 410,000, and 600,000 Da). During the experiment, the column was kept at 40 °C. The molecular weights of ACP1-1 and ACP2-1 were estimated by reference to a calibration curve constructed using the dextrans of known molecular weights.

### 3.4. Monosaccharide Composition of ACP1-1 and ACP2-1

Gas chromatography (GC) was used for identification and quantification of the monosaccharide in ACP1-1 and ACP2-1. First, ACP1-1 and ACP2-1 were hydrolyzed with 2 M trifluoroacetic acid (TFA) (2 mL) at 120 °C for 2 h. Excess acid was completely removed by distilled water. Then, the hydrolyzed products were mixed with 2 mL of pyridine, immediately followed by 0.4 mL of trimethylchlorosilane, and 0.8 mL of hexamethyldisilazane. The mixture was shaken in a 50 °C water bath for 15 min to dissolve the solute. Then, 1.5 mL of deionized water was added. After the solution had separated out, the supernatant was isolated by centrifugation at 3000 rpm for 10 min. Standards (arabinose, rhamnose, ribose, xylose, mannose, galactose, and glucose) were also prepared in the same way and subjected to GC analysis separately.

### 3.5. UV/Vis and FT-IR Analysis

The impurity content of the polysaccharide was determined in quartz colorimetric utensil using UV/Vis spectroscopy at an optical path length of 1 vm and a scan interval of 1 nm. The spectrum of ACP1-1 and ACP2-1 (1 mg/mL) were recorded in the region 200–400 nm at 25 °C. Organic functional groups and the primary structure of polysaccharide were identified according to the spectrum of FT-IR compared with previous studies. For FT-IR, ACP1-1 and ACP2-1 (3 mg) were dried at 35–45 °C under vacuum, then ground to a powder with spectroscopic grade KBr. The powder was pressed into a 1 mm pellet. Spectra were recorded from 400 to 4000 cm^−1^ with a resolution of 8 cm^−1^ resolution and 32 scans at 25 °C. The spectrum performed a smoothing and a correction of the baseline by using Origin 8.6 software.

### 3.6. Periodate Oxidation

The locations of glycosidic linkages in polysaccharides can be preliminarily determined by periodate oxidation, which involves consumption of periodate and production of formic acid [57]. ACP1-1 and ACP2-1 (10 mg of each) were oxidized with 0.15 M NaIO_4_ (40 mL) and kept in the dark. The absorption was monitored at 223 nm every 4 h. Complete oxidation, identified by a stable absorbance, was reached in 96 h, and excess NaIO_4_ was removed at this time by adding ethylene glycol. Consumption of NaIO_4_ was measured by a spectrophotometric method, and formic acid production was determined by titration with 0.005 M NaOH.

### 3.7. Methylation Analysis

ACP1-1 and ACP2-1 (10 mg of each) were methylated three times according to the method of Needs and Selvendran [58]. The methylated products were extracted into chloroform and examined by FT-IR. The absence of a hydroxyl absorption peak indicated methylation was complete. The methylated products were hydrolyzed with formic acid and 2 M TFA for about 2 h, and excess acid was removed by co-distillation with distilled water or methanol. Each hydrolysate was combined with 2 mL of acetic anhydride and 2 mL of pyridine and heated at 100 °C for 1 h. After acetylation with acetic anhydride, product was analyzed by gas chromatography-mass spectrometry (GC-MS) on an GC 7890 N gas chromatograph (Agilent, USA) coupled with an Agilent 5973 N mass-selective detector. The GC-MS conditions were as follows: The GC capillary column was DB-1701 (0.25 mm × 30 m, 0.25 mm) and the mass scan range was 30–450 *m*/*z* (electron ionization 70 eV). The injector and detector were operated at a temperature of 220 °C and 280 °C, respectively. Temperature program was programmed as follows: initial temperature of 150 °C was held for 3 min and then increased to 260 °C by 15 °C/min and held for 5 min. And helium was used as the carrier gas with a constant flow rate of 1 mL/min.

### 3.8. Statistical Analysis

Statistical Product and Service Solutions (SPSS) and Origin 8.6 software were used to analyze the results. The data are reported as means ± standard deviations. The differences between groups were analyzed using one-way analysis of variance (ANOVA), and correction for multiple comparisons was made through a Dunnett’s multiple comparison test. Differences were considered significant at *p* < 0.05.

## 4. Conclusions

Two novel water-soluble polysaccharides were isolated from *A. cordifolia* and characterized. The trichloroacetic acid method was the most effective approach to remove the protein among Sevag method, trichloroacetic acid method, and hydrochloric acid method. X-5 resins exhibited higher polysaccharide retention rates and decolorization rates than those of the other four resins tested. Based on the monosaccharide composition, methylation analysis, periodate oxidation, and FT-IR data, we determined the main chain of ACP1-1 contains (1→3,6)-galacturonopyranosyl residues interspersed with (1→4)-glucopyranosyl residues and (1→3)-mannopyranosyl residues. The main chain of ACP2-1 consists of (1→3)-galacturonopyranosyl residues interspersed with (1→4)-gluco-pyranosyl residues. This information could be used to investigate the mechanisms underlying food-based therapies and be used to develop health care products. For further studies, NMR spectroscopy should be employed to illustrate the structures of ACP1-1 and ACP2-1 in more details and relevant biological activities of ACP1-1 will be explored.

## Figures and Tables

**Table 1:** Deproteinization effects by different methods in ACP.

Effect	Trichloroacetic Acid	Sevag	Hydrochloric Acid
Protein clearance rate/%	97.73 ± 0.52	97.31 ± 0.56	55.10 ± 0.43
Polysaccharide loss rate/%	4.86 ± 0.37	49.67 ± 0.26	13.53 ± 0.35

**Table 2:** Decolorization effects by different methods in ACP.

Resin	Polarity	Decolorization Rate/%	Polysaccharide Retention Rate/%
D101	Apolar	55.24 ± 0.24	59.19 ± 0.44
X-5	Apolar	72.16 ± 0.47	75.24 ± 0.17
AB-8	Week polar	59.13 ± 0.32	54.43 ± 0.29
HPD-400	Week polar	43.86 ± 0.31	65.76 ± 0.31
NKA-9	Polar	45.59 ± 0.28	68.38 ± 0.23

**Table 3:** Methylation analysis of ACP1-1.

Methylated Sugar	Type of Linkage	Mass Fragments (*m*/*z*)	Molar/%
2,4,6-Me3-Gal	→3-Gal*p*-(1→	58, 87, 101, 117, 149, 161, 203	5.02
2,3,6-Me3-Glc	→4)-Glc*p*-(1→	87, 101, 117, 143, 161	38.12
3,6-Me2-Man	→2,4)-Man*p*-(1→	87, 99, 113, 129, 159, 203, 233	2.49
2,3,4,6-Me4-Glc	Glc*p*-(1→	71, 87, 101, 117, 129, 145, 161, 205	1.30
2,4,6-Me3-Man	→3)-Man*p*-(1→	87, 129, 159, 173, 187	1.12
2,3,4-Me3-Gal	→6)-Gal*p*-(1→	71, 87, 117, 161	1.52
2,4-Me2-Man	→3,6)-Man*p*-(1→	58, 87, 117, 129, 159, 189	13.31

**Table 4:** Methylation analysis of ACP2-1.

Methylated Sugar	Type of Linkage	Mass Fragments (*m*/*z*)	Molar/%
2,4,6-Me3-Gal	→3)-Gal*p*-(1→	71, 87, 101, 117, 129, 161	1.10
2,3,6-Me3-Glc	→4)-Glc*p*-(1→	71, 87, 117, 129, 161, 173	9.87
